# *In silico* trials: Verification, validation and uncertainty quantification of predictive models used in the regulatory evaluation of biomedical products

**DOI:** 10.1016/j.ymeth.2020.01.011

**Published:** 2021-01

**Authors:** Marco Viceconti, Francesco Pappalardo, Blanca Rodriguez, Marc Horner, Jeff Bischoff, Flora Musuamba Tshinanu

**Affiliations:** a Department of Industrial Engineering, Alma Mater Studiorum – University of Bologna, Italy; b Medical Technology Lab, IRCCS Istituto Ortopedico Rizzoli, Bologna, Italy; c Dipartimento di Scienze del Farmaco, University of Catania, Italy; d Department of Computer Science, British Heart Foundation Centre of Research Excellence, University of Oxford, UK; e ANSYS, Inc., Evanston, IL, USA; f Corporate Research Department, Zimmer Biomet, Warsaw, IN, USA; g Federal Agency for Medicines and Health Products, Brussels, Belgium

**Keywords:** *In silico* trials, Regulatory affairs, Model credibility, Verification, Validation

## Abstract

Historically, the evidences of safety and efficacy that companies provide to regulatory agencies as support to the request for marketing authorization of a new medical product have been produced experimentally, either *in vitro* or *in vivo*. More recently, regulatory agencies started receiving and accepting evidences obtained *in silico*, i.e. through modelling and simulation. However, before any method (experimental or computational) can be acceptable for regulatory submission, the method itself must be considered “qualified” by the regulatory agency. This involves the assessment of the overall “credibility” that such a method has in providing specific evidence for a given regulatory procedure. In this paper, we describe a methodological framework for the credibility assessment of computational models built using mechanistic knowledge of physical and chemical phenomena, in addition to available biological and physiological knowledge; these are sometimes referred to as “biophysical” models. Using guiding examples, we explore the definition of the context of use, the risk analysis for the definition of the acceptability thresholds, and the various steps of a comprehensive verification, validation and uncertainty quantification process, to conclude with considerations on the credibility of a prediction for a specific context of use. While this paper does not provide a guideline for the formal qualification process, which only the regulatory agencies can provide, we expect it to help researchers to better appreciate the extent of scrutiny required, which should be considered early on in the development/use of any (new) *in silico* evidence.

## Highlights

• Regulators now consider also evidences produced *in silico*.
• We need accepted methods to evaluate the credibility of models.
• In this paper we describe the use of the ASME V&V-40 technical standard.
• We also discuss its application to various types of modelling methods.

## 1 Introduction

Modelling and simulation are standard practice in many industrial sectors as support to the design and the de-risking (intended as the evaluation of safety and performance) of new products. Computer modelling and simulation of humans in both health and disease is a powerful tool in biomedical research, augmenting experimental and clinical research through detailed mechanistic and systematic investigations which are impossible with other means [1], [2]. A large body of research across biomedicine has expanded the credibility of modelling and simulation beyond academia, with dynamic activity also in regulatory agencies and industry [3], [4], [5]. Thus, human *in silico* clinical trials are now emerging as an important paradigm in the development of medical therapies [6]. This class of trial exploits human-based modelling and simulation technologies for virtual testing of pharmacological therapies [7] and devices [8]. In addition, modelling and simulation is being used to reduce, refine and replace animal experimentation [9], [10], and even to replace bench tests [11], [12]. Taking this broad range of applicability into account, the term “in silico trials” refers to the use of modelling and simulation in both the preclinical and clinical evaluation of a new medical product.

A number of organizations formed in the past decade were critical to enabling the use of modelling and simulation to develop the *in silico* trials concept, primarily for medical devices. For example, the American Society of Mechanical Engineers (ASME) VV-40-2018 “Verification and Validation in Computational Modeling of Medical Devices” technical committee was established by the ASME Division of Codes & Standards in 2010. In 2012, the Medical Device Innovation Consortium was formed as a public-private partnership between the US Food & Drug Administration (FDA), the Center for Medicare & Medicaid Services (CMS), the National Institutes of Health (NIH), the medical device industry, not-for-profit organizations, and patient associations. One of the first projects of this organization was the Computer Modeling and Simulation Project, aimed to balance the desire for certain device performance with the need to reduce the delay in patient access, using modelling and simulation as valid scientific evidence. In 2013, the European Commission launched a Support Action named “Avicenna: A Strategy for In-Silico Clinical Trials”, that saw the participation of over 600 experts from academia, industry, regulatory agencies, and patient organisations. One goal of Avicenna was the elaboration of a public research roadmap on the adoption of modelling and simulation for regulatory purposes [13]. Thanks to these initiatives, between late 2015 and 2016 both the US Congress and the European Parliament made similar recommendations toward their respective regulatory agencies (FDA in the USA, and The European Medicines Agency, or EMA, in Europe) stressing the need to adopt *in silico* assessment as part of the regulatory process. In 2016, the FDA Center for Devices and Radiological Health (CDRH) published a first guidance on “Reporting of Computational Modeling Studies in Medical Device Submissions”.^1^ This was followed in 2018 by the publication of the ASME V&V 40-2018 technical standard “Assessing Credibility of Computational Modeling through Verification and Validation: Application to Medical Devices”.^2^

Mechanistic *in silico* modelling is also rapidly evolving in the pharmaceutical area. For example, specific to disease modelling, human cardiac electrophysiology is one of the most advanced areas in physiological modelling and simulation. Current human cardiac electrophysiology models integrate detailed information on the dynamic processes underlying cardiac electrical excitation from subcellular to whole organ levels [14]. Utilizing these frameworks, modelling and simulation studies have played a central role in the discovery of cardiac arrhythmia mechanisms [15], [16] and treatments such as electrical defibrillation [17].

Building on a high level of model maturity, in 2013 the Comprehensive *in vitro* Proarrhythmia Assay (CiPA) initiative was proposed as a new strategy for the assessment of the pro-arrhythmic risk of pharmaceutical compounds for regulatory purposes, and was sponsored by the FDA, the Cardiac Safety Research Consortium (CSRC), and the Health and Environmental Science Institute (HESI). CiPA has become a global effort, involving many industry and academic partners, in addition to regulators [18], [3]. The main novelty proposed by CiPA was the adoption of modelling and simulation for the characterization of the torsadogenic effects of drugs, which is currently mostly ensured by *in vitro* testing (herG assay), animal *in vivo* and clinical trials. Specifically, CiPA proposed *in silico* analysis of human ventricular electrophysiology using high-throughput *in vitro* screening of drug effects on multiple human ion channels for safety assessment of new pharmaceutical compounds. This triggered ongoing inter-sectoral collaborations to define the standards required for the qualification of models and simulations for CiPA, to identify and incorporate new technologies for clinical and non-clinical applications, including refinement of *ex vivo* and *in vitro* assays and screens, *in vivo* models, non-invasive clinical modalities, and *in silico* approaches.

As *in silico* methods are increasingly included in regulatory submissions, it is the authors’ opinion that the research community should agree on a certain level of scrutiny to be considered as a minimum requirement when reporting *in silico* results either in peer-reviewed publications or in regulatory submissions. To this end, one aim of this paper is to provide an introduction to the model credibility evaluation process introduced by the ASME VV-40-2018 standard. The paper will also compare and contrast this evaluation process with a recent EMA guideline on the reporting of physiologically based pharmacokinetic (PBPK) modelling and simulations, which is the first guideline on patient-specific modelling and simulation published by the EMA^3^ and shares key features with the ASME VV-40-2018 standard. Both sources provide both a step-by-step overview of the credibility assessment of predictive models that can be used to inform the planning, implementation and assessment of *in silico* analyses, and the minimum requirements for model qualification given the context of use and the regulatory impact. We will also discuss possible generalizations to other modelling techniques and contexts of use.

## 2 The ASME V&V 40 credibility assessment process

**Fig. 1:**
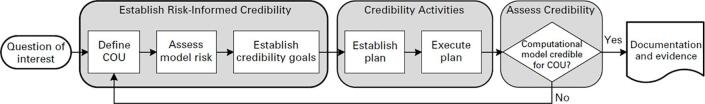
The risk-informed credibility assessment framework of ASME V&V40-2018 (reprinted from ASME V&V 40-2018 by permission of the American Society of Mechanical Engineers. All rights reserved).

The ASME VV-40-2018 standard, ‘Assessing Credibility of Computational Modeling through Verification and Validation: Application to Medical Devices’, introduced the risk-informed credibility assessment framework shown in Fig. 1. The credibility assessment process begins with a question of interest, which is generally framed around a specific aspect of the functional performance of a medical device that is linked to its safety and/or efficacy. In practice, the question of interest can be answered with data generated through a variety of pre-clinical (or clinical) experiments. For those questions that may be addressed either entirely or in part with modelling and simulation, the ‘Context of Use’ (COU) is the term used by the standard to specify the role of modelling and simulation in addressing the question of interest. These two terms (question of interest and COU) will be described in more detail in Section 2.1. With a well-defined COU, the model risk can be identified (Section 2.2); though the concept of model risk is not novel to this standard, it takes an important role in this discussion because of the potential impact of biomedical products (including devices) on human health and safety. The model risk is then used to establish credibility goals for the computational model (Section 2.3) that can be achieved through careful planning and execution of model verification (Section 2.4) and validation (including uncertainty quantification) (Section 2.5) activities. By evaluating the applicability of the verification and validation activities to the COU (Section 2.6), again mindful of the model risk, an assessment of whether there is sufficient model credibility to support the COU can be made. Each of these key steps will be described here in more detail.

### 2.1 Definition of the question of interest and context of use

As shown in Fig. 1, the risk-informed credibility process begins by identifying a question of interest. The question of interest describes the specific question, decision or concern that is being addressed with a computational model. In other words, the question of interest lays out the engineering question that is to be answered (at least in part) based on a model. The next step is to define the context of use (or COU), which establishes the specific role and scope of the model in addressing the question of interest. The COU provides a detailed and complete explanation of how the computational model output will be used to answer the question of interest. The COU should also include a description of the other sources of evidence that will be used as part of the decision, such as data from bench testing animal and human trial data, and/or historical (registry) data.

### 2.2 Risk analysis

**Fig. 2:**
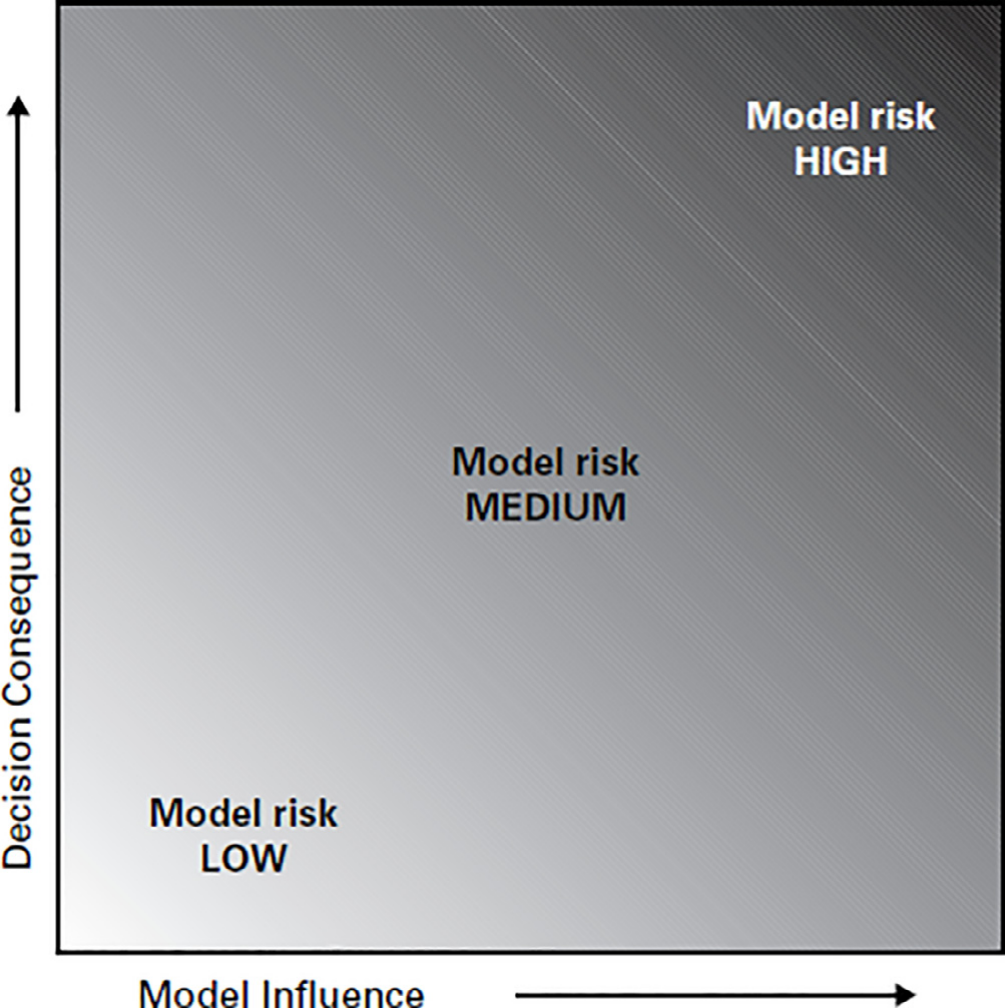
The risk assessment matrix of ASME V&V40-2018 (reprinted from ASME V&V 40-2018 by permission of the American Society of Mechanical Engineers. All rights reserved).

The next step is to determine the model risk, which represents the possibility that the model may lead to false or incorrect conclusions, potentially resulting in one or more adverse outcomes. Examples of adverse outcomes include a poor result for the patient, the need for re-intervention by a clinician, or loss of company revenue or reputation. As shown in Fig. 2, model risk is defined as a combination of model influence and decision consequence, where:

- Model influence represents the contribution of the computational model to the decision in relation to other available evidence. The relative contribution of the device safety/efficacy evidence sourced from a computational model increases from left to right on the *x*-axis of Fig. 2. This element of risk is explicitly tied to the COU since that is where the other sources of evidence used to make a decision about device safety/efficacy are established.
- Decision consequence refers to the significance of an adverse outcome resulting from an incorrect decision, i.e. the “severity” of the adverse outcome if the decision based on the model is incorrect. While decision consequence is not explicitly linked to the risk classification of a device (i.e. Class I, II, or III), it follows that Class III devices (representing the highest risk device classification in the US) are more likely to rely on high-risk models versus Class I (i.e. low risk) devices. This is reflected in the decision consequence because the severity of the incorrect decision is manifested here.

Model risk is a combination of model influence and of decision consequence and reflects the risk associated with making a decision based, at least in part, on the output of a computational model.

### 2.3 Establish credibility goals

Having identified the overall model risk, the next step in the risk-informed credibility framework is to establish the credibility requirements for the computational model. As mentioned previously, model credibility refers to the trust in the predictive capability of a computational model for a specific COU, where trust is established through the collection of verification and validation (V&V) evidence and by demonstrating the applicability of those V&V activities to support the model for the COU. Therefore, the objective of this step is to determine a set of requirements such that the computational model has *sufficient* credibility for the COU. To assist in this process, the ASME V&V 40 standard defines a set of credibility factors, which represent the constituent elements of a credibility evaluation process. The user evaluates each of these factors and must demonstrate that they will be able to collect enough evidence such that the overall credibility of the computational model is commensurate with the model risk.

Activities associated with establishing the credibility of a computational model can be sub-divided into three categories: verification, validation (including uncertainty quantification), and applicability. The credibility factors identified in the V&V 40 standard fall into these three categories, which are briefly described in the remainder of this section. The reader is referred to ASME VV-40-2018 for a complete description of all credibility factors and to ASME VV-10-2007,^4^ ASME VV-20-2009,^5^ Oberkampf and Roy [19], and Roache [20] for more detailed information on model verification, validation, and uncertainty quantification.

### 2.4 Verification

The goals of verification are to ensure that the computer model and simulation framework provides a faithful representation of the intended mathematical model and its solution, and to quantify the errors present in the numerical solution of the mathematical model. The two elements of verification are code verification and calculation verification. Code verification aims to identify errors in the source code and numerical algorithms of a code platform, while calculation verification aims to estimate the magnitude of the numerical errors in the discrete solution (e.g. discretization errors and iterative errors). Therefore, verification provides mathematical evidence regarding the accuracy of a numerical solution.

#### 2.4.1 Code verification

Code verification provides assurance that a platform is free of bugs in the source code and numerical algorithms. This form of verification relies on comparing the output from a specific code platform to benchmark problems with known analytical solutions. Calculating an observed order of convergence is the most stringent form of code verification and is determined by calculating the rate of convergence of the solution on a sequentially refined series of meshes [6]. The code is considered verified if the observed order of convergence agrees with the theoretical convergence rate, e.g. the element order in finite element analysis (FEA) or order of the up-winding scheme in computational fluid dynamics (CFD). Other (less stringent) code verification methods include determination of the discretization error as part of a mesh refinement study or comparison to the results of a previously verified code.

#### 2.4.2 Calculation verification

Calculation (or model) verification can be initiated once the user has reasonable assurance of the reliability of the code platform. The objective of calculation verification is to estimate the error in the output of a computational model due to the use of numerical methods to solve the mathematical model specific to the COU. In contrast to code verification, an exact solution is not required (indeed, why perform modelling if an exact solution were in fact available?). In calculation verification, the spatial and temporal convergence behaviour is analysed and quantified by refining the discretization in both space and time. While the focus of calculation verification is typically on discretization errors, other sources of error include round-off error, numerical solver (iterative) error, and user error. It is safe to proceed to the validation process once errors in the numerical solution have been demonstrated to have been minimized to the point that they are not polluting the numerical results.

### 2.5 Validation

Validation is the process of assessing the degree to which a computer model and simulation framework is able to simulate a reality of interest. Put another way, validation activities are concerned with demonstrating the correctness of the underlying assumptions that were used to guide the development of the mathematical model. This goal is accomplished by developing a validation comparator, which provides the data used to evaluate the output of simulations using the computational model. It is important to note that there is no validation in the absence of comparator data. Two validation metrics are used to establish model credibility when making this comparison: one is the difference between the simulation and the comparator outputs and the other is an estimate of the uncertainty in this comparison.

#### 2.5.1 Model/comparator/assessment

As a comparative process, appropriate validation activities require attention to both the computational model and the comparator, with an appropriately rigorous evaluation of both the experimental and simulation procedures. This includes the development of a validation comparator whose performance and outputs mirror the behaviour of the mathematical model as closely as possible. An evaluation of the control parameters (model inputs) of the comparator and measured values (model outputs) is also required. Only through careful construction of the comparator will the data required to establish model credibility be obtained.

Validation also helps to ensure that the computational model has sufficient rigor for the intended context of use. This level of rigor is established through an assessment process that considers the equivalency of the input and output parameters of the simulation and comparator. Generally, model credibility increases when inputs and outputs are equivalent, the rigor of the comparison is as high as possible, and the level of agreement between model and comparator is high. It is important to note that a more rigorous or more precise model is not necessarily more credible; for example, more complex models usually require more parameters to inform them, some of which might be affected by considerable uncertainties, and may confound discovery and resolution of basic errors in model form. Instead, the focus of the V&V process should be on creating a model with *sufficient* rigor, which will result in the optimal use of model development resources as well as simulation infrastructure.

#### 2.5.2 Uncertainty quantification

A second element of validation is the degree to which sensitivities and uncertainties of the computational model and the associated comparator(s) are understood. The three sources of computational model uncertainty are uncertainties due to modelling assumptions and approximations, uncertainties resulting from the numerical solution of the governing equations, and uncertainties in the model input parameters. When combined with uncertainties in the experimental results, these provide insight into what adjustments in the model form will potentially improve agreement between simulation and experiment. The uncertainty metric is essential when assessing the credibility of higher risk models. For example, a model that agrees with experimental results but has high uncertainty in model form is suspect since the high uncertainty undermines the credibility of the model when making predictions. Similarly, a model exhibiting poor agreement with experimental data and low uncertainty provides little insight into what element(s) of the model form (or the comparator) can be addressed to improve the agreement. ASME VV-20-2009 and other texts [19], [20] provide significant background on this topic.

### 2.6 Applicability

**Fig. 3:**
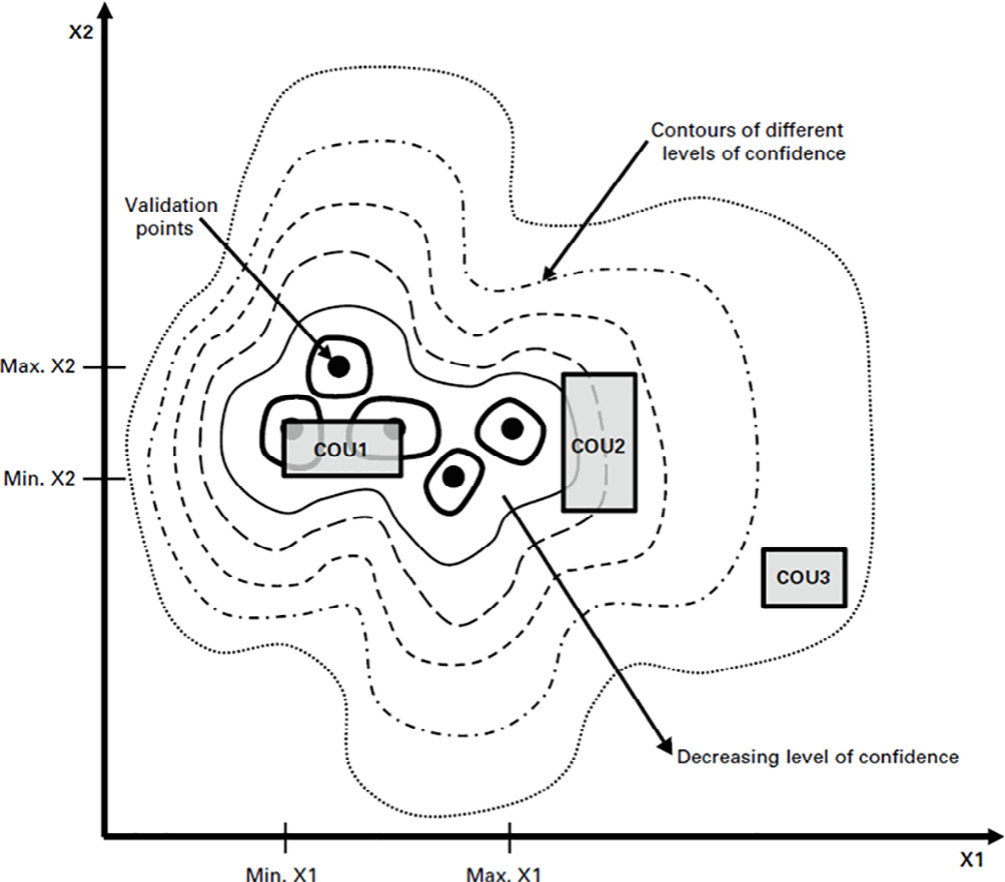
Schematic representation of applicability (reprinted from ASME V&V 40-2018 by permission of the American Society of Mechanical Engineers. All rights reserved).

The applicability of a computational model refers to the relevance of the validation activities to the COU and is represented schematically in Fig. 3. There is typically an assortment of model input parameters (e.g. X1 and X2 in Fig. 3) that may be either variable (e.g. blood pressure, bone stiffness) or parametric (e.g. device size, device material). Applicability refers to the range for the model input parameters that characterize or bound the validation activities, and which also position the COU relative to the validation activities. Qualitatively, the closer the validation activities are to the COU in terms of these key parameters, the more confidence there is in the predictive capability of the model. An additional aspect of applicability is the extent to which a quantity of interest (QOI) of the COU is linked to the measurements and model predictions from the validation activities. Validation activities are limited to aspects of device performance which can be directly measured within a physical setting, whereas each QOI of the COU may be more deeply embedded (and essentially unmeasurable) within the biophysical system. In addition to addressing potential gaps between input system parameters, assessing the applicability also requires careful consideration of the gap between the measured validation outputs and each QOI [21]. Pathmanathan et al. proposed a step-by-step applicability analysis that can facilitate the identification of gaps between validation evidence and a COU [22].

### 2.7 Clinical applicability

The ASME VV-40-2018 credibility framework can be viewed as a thoughtful process to guide careful planning, execution, and analysis of model verification and validation activities. Such activities necessarily entail consideration of model predictions relative to data from other sources, such as benchtop testing and animal studies. In some cases, the data may also come from clinical studies on the same biophysical product, e.g. a reduced but representative clinical cohort. In many cases however, the data come from studies that are several steps removed from the intended clinical scenario. For example, validation of a model to predict the potential for bone remodelling around a hip stem may utilize strain gage or digital image correlation measurements on a synthetic bone that is loaded in a controlled benchtop experiment, whereas the COU for that model is intended to capture the range of anatomies, bone tissues, and *in vivo* loading conditions encountered in clinical practice. For these cases, extension of the validation model to the COU then requires modification of key input parameters. For example, the relatively well understood mechanical properties of the synthetic bone are replaced by the range of tissue stiffnesses and inelasticities that are encountered in total hip replacement patients. Similarly, the small number of discrete loading scenarios that are tested in the lab as part of the model validation activities, and which presumably resulted in adequate predictions of strain on the surface of the synthetic bone, are replaced by an assumed spectrum of loading conditions that characterize loading of the hip across a range of different activities of daily living. The appropriate specification of these parameters, intended to represent the functional range of the biophysical environment in which the device or product is expected to reside is critical to the effective use of the model, but may be incorrectly specified for an otherwise credible model. Continuing the above example, the user may have misstated the bounds of bone density, or incorrectly estimated the number of steps that a hip replacement patient may take in a year. These misstatements of the COU may result in incorrect conclusions being drawn from a model that was otherwise shown to be credible based on the validation activities. In other words, one might have developed (and deployed) a model which is credible for a COU but has minimal clinical applicability due to a misunderstanding of the intended clinical environment. Returning back to the schematic representation of Fig. 3 – assessing applicability per ASME VV-40-2018 will address the relevance of validation activities to COU1, for example. However, it will not ensure that the analyst properly identified COU1 in the first place; in fact, maybe COU3 should have been the clinical target.

## 3 Extension to other types of models

The broad research field of modelling and simulation in biomedicine involves the use of a variety of mathematical and statistical approaches. In addition to physics- and chemistry-based mechanistic models, developed from reliable first principles formulated in term of field theories and differential equations, the literature is full of interesting studies using discrete modelling approaches such as agent-based modelling, semi-mechanistic approaches such as Bayesian modelling, and fully phenomenological, data-driven approaches, such as machine learning. This section evaluates the suitability of the V&V 40-2018 standard outside its stated scope of physics-based models. This is intended as a starting point, as an in-depth evaluation of each model type is beyond the scope of this paper.

### 3.1 The implicit assumptions for physics-based models

The ASME VV-40-2018 standard was developed with a fairly specific, although very popular, class of modelling methods in mind, namely physics-based, mechanistic models. What are the cautions required if the model to be used does not fall into that class?

Let us assume that a generic predictive model can be represented mathematically as:

(1) $$\hat{O}=f\left(I\right)$$

The predictive error of the model *f()* can be described as:

(2) $$\left|O-\hat{O}\right|=\alpha_{I}+\varepsilon_{f}+\nu_{f,I}$$

where $\alpha_{I}$ is the *aleatoric error* due to the uncertainty affecting the observational data used to inform/build the model, $\varepsilon_{f}$ is the *epistemic error*, due to inability of the mechanistic knowledge used to build the model (if any) to reproduce completely the physical reality being modelled, and $\nu_{f,I}$ is the *numerical approximation* error that occurs when solving the mathematical forms that represent the mechanistic knowledge in an approximated way.

Verification, validation, and uncertainty quantification studies rely on some typical assumptions about the nature and form of these three errors. One can see where the limits of these assumptions are by making them explicit. The first assumption is that the distribution of $\alpha_{I}$ over repeated measurements has null mean. If this is true for a sufficiently large number of validation experiments, then we can write:

(3) $$\mathrm{ave}\left(\left|O-\hat{O}\right|\right)\approx \varepsilon_{f}+\nu_{f,I}$$

If $\alpha_{I}$ has zero mean, the average predictive error over a sufficiently large number of validation experiments should depend only on the mechanistic and numerical approximation errors. Thus, the first check is to ensure that all the input data fed into the model are not affected by significant systematic errors.

The second assumption is that the error due to the numerical approximation is much smaller than that due to the epistemic uncertainty. Under this assumption:

(4) $$\mathrm{ave}\left(\left|O-\hat{O}\right|\right)\approx \varepsilon_{f}$$

In other words, verification is aimed to confirm that $\varepsilon_{f}\gg \nu_{f,I}$ so that validation can provide an estimate of the epistemic error.

Next, we assume that all the variability affecting the predictive error is only due to the aleatoric component, and thus:

(5) $${\mathrm{var}(\left|O-\hat{O}\right|)\approx \alpha }_{I}$$

Thus, the uncertainty affecting the predictive error can be quantified by computing how the uncertainty affecting the inputs propagates to the outputs.

The last assumption relates to the applicability concept outlined in the V&V 40 standard. We assume that $\varepsilon_{f}$ is fairly constant (and small) for an ample range of input values, and then starts to degrade with a certain degree of smoothness. This is fairly true for most physics-based models: for example, in a model that assumes the material is a linear elastic solid, the epistemic error due to this assumption will be fairly constant and low below the yield point, and will increase smoothly as the stresses increase beyond the yield limit.

### 3.2 Statistical and machine learning models

Predictive models developed using traditional frequentist statistics [23], [24], [25], as well as machine learning models [26], [27], [28], do not rely in general on prior knowledge; thus, they are not affected by epistemic error. Also, there are no numerical approximation errors since there is no mathematical model to be solved numerically. On the other hand, in general we cannot make any assumptions regarding the statistical distribution of the aleatoric error, which might have, for example, a non-normal distribution and a non-null average. Thus, the credibility of these models needs to be assessed using different approaches. At risk of oversimplifying, the credibility of these models can only be assessed by induction, and thus these models are never truly validated. Indeed, a recent FDA proposal outlining a regulatory framework for Artificial Intelligence/Machine Learning (AI/ML)-Based Software as a Medical Device (SaMD) [29] suggested that these models should be continuously tested in order to avoid issues such as context drift [30].

### 3.3 Bayesian models and grey-box models

In Bayesian models [31], [32], [33], the posterior probability is the sum of the prior probability (which if informed by *a priori* knowledge can be assumed to affect primarily the epistemic error) and the likelihood (which is informed by observational data, and thus is affected by aleatoric errors). Grey-box models are a very broad category that include a variety of modelling methods. A good example is the Nonlinear AutoRegressive Moving Average with eXogenous input (NARMAX) model [34], [35]. In general, these methods rely on mathematical models that are able to explain mechanistically only part of the phenomenon, using an entirely phenomenological model for the remaining system behaviour(s).

The credibility approach described in V&V 40 can still be useful for these types of models. However, because of the nature of these models, it is quite difficult to make any hypothesis on how the epistemic error varies with the model inputs. Thus, consideration of the applicability of validation activities to the question of interest (which may utilize a different range of model inputs) can be challenging to defend. As a first educated guess, we would recommend to never trust any prediction made outside the range of validated inputs and to design validation studies with a significant number of points in the input space.

### 3.4 Agent-based models

Agent-based models (ABM) are an effective approach for modelling discrete, autonomous agents such as cells or bacteria. It is quite common to model cancer [36], [37], [38] and immune-related diseases [39], [40], [41], among others using ABM. The credibility assessment of ABM is a complex topic; some extensive discussion can be found here [42]. However, the assumptions of conventional verification, validation and uncertainty quantification processes are potentially valid for ABM, and thus the V&V 40-2018 standard can be readily applied to these models.

## 4 Application to pharmaceutical, biological, or combinatory products

### 4.1 Overview

The V&V 40-2018 standard specifically refers to the application to medical devices. Does this limit the applicability of the standard to assess the credibility of a computer model designed to evaluate the safety and/or the efficacy of a new medicinal, biological, or combinatory product? Strictly speaking, the answer should be no, but since there is currently no technical standard available for these other purposes, it is worthwhile to explore this topic in more detail.

Combinatory products are, according to the FDA, products “composed of any combination of a drug and a device; a biological product and a device; a drug and a biological product; or a drug, device, and a biological product”.^6^ However, the most common instance is a medical device that releases some pharmaceutical substance as part of its function; a good example are drug-eluting stents [43]. For most of these devices, the active substance being released is already in clinical use, so the most critical aspect is the drug release kinetics, which can be described with biophysical models. In fact, these products follow a regulatory pathway that is fairly similar to that of medical devices. Thus, it is reasonable to assess the credibility of the model with the V&V 40 standard.

Biological and medicinal products follow very different regulatory pathways, however. Instead of using technical standards, it is recommended that, if new methodologies are used to inform the characterization of safety and/or efficacy of a (new) product, they undergo a regulatory process on their own, known as “qualification”. FDA calls it the Drug Development Tools Qualification program,^7^ while EMA calls it the qualification of novel methodologies for medicine development.^8^

Historically, qualification focused on experimental or (pharmaco)statistical methods, such as population pharmacokinetics (popPK), pharmacokinetic-pharmacodynamics (PK/PD), and dose-exposure response (DER) models [44]. However, it should be noted that so far most of these models (popPK, PK/PK and DER) tend to be simpler from a mathematical and numerical point of view as compared to those discussed in 2, 3. One reason is that these models aim at predicting the average behaviour of a population of patients rather than the behaviour of an individual patient, and parameter estimations from clinical data is most frequently part of the model development process. For these models, predictive error is therefore driven by different considerations.

As far as more mechanistic PK models are concerned, both FDA and EMA have released guidelines^9^^,^^10^ for the qualification of physiologically based pharmacokinetics (PBPK) models, where model verification and sensitivity analysis are also required. As more complex and sophisticated models are submitted for regulatory qualification, we see some value for the scientists involved in model development and assessment to consider the criteria included in the V&V 40 standard and the EMA PBPK guideline (as detailed in the next section), as long as for the problem at hand, the assumptions made in section 3.1 are acceptable. In addition, it seems almost mandatory to include a clinical validation, where the model prediction is compared to the clinical observation in an adequate number of patients/time points in addition to an extensive technical validation based on controlled experiments performed *in vitro*, *ex vivo*, or *in vivo*.

### 4.2 Qualification of platforms and drug models according to the EMA PBPK guideline

The EMA PBPK guideline provides recommendations for PK model characterization, but interestingly, the indications for assessing the quality of these mechanism-based models have various points in common with the ASME V&V 40 standard.

In the guideline, similar to what is described in the V&V 40 document, the context of use and the so-called regulatory impact are considered important starting points of consideration for model assessment. Model qualification also depends on assessment criteria very similar to those described in the ASME V&V 40 standard. They include qualification of the platform (system model) used, verification of model equations and input parameters, characterization of assumptions, and uncertainties, sensitivity analyses and assessment of model predictive performances.

In addition to the description of the context of use, the guideline recommends specifying the regulatory impact of the model, which is of utmost importance when determining the qualification requirements. The regulatory impact is directly linked to the risk to the patient in case the modelling predictions or assumptions lead to erroneous regulatory decisions. The impact of a simulation also depends on how much weight of evidence the model-based simulation will have in certain scenarios, the therapeutic context, and the resulting treatment recommendations. These risk considerations directly correspond to the VV-40-2018 risk elements of decision consequence and model influence, respectively. Regulatory impact can be classified as high, moderate or low, where the qualification requirements increase with regulatory impact.

Parameters related to human patho-physiology (referred in the guideline as *system-depended parameters*) need to be defined with particular attention. Reliability of the sources of drug-related and system-related model input parameters is considered important and references are to be provided. Additionally, the rationale for the chosen system-dependent parameter values should be given.

Data to support the assumptions and their biological and/or pharmacological rationale should also be presented and discussed, as well as the impact of those assumptions on the model and the outcome. It is recommended that a sensitivity analysis be undertaken for key parameters (i.e. ones that are likely to markedly influence the outcome) or parameters that are uncertain.

As is the case for ASME VV-40-2018, model verification is the part of the qualification process that is focused on the assessment of the correctness of the mathematical model structure, including details of the differential equations used and the parameterisations of the model.

In general terms, the qualification report for a particular context of use should show the ability of the model to predict observed outcomes, what in other contexts is referred to as validation. The search strategy for the *in vivo* studies included to support the intended use of the platform (systems model) should be shown and justified.

### 4.3 *In silico* augmented clinical trials

An important question remains open: can modelling and simulation be used to reduce the human experimentation required in the regulatory process? In an increasing manner, pharmacometrics models such as population PK, PK/PD, E/R and PBPK models are used by pharmaceutical companies and endorsed by the regulatory agencies to replace clinical trials in the context of drug approval for various applications. Different types of studies are now routinely replaced by modelling and simulation evidence under well-defined conditions. They include (but are not limited to): PK-related drug-drug interactions studies, therapeutic studies for small populations (e.g. children, rare disease), for new pharmaceutical formulations and biosimilars, cardiac safety (QTc prolongation) studies, dose finding studies, etc. For patient-specific computer models, this is currently an area of intense regulatory science research. A recent paper from some of the authors tries to frame this into a proper theoretical framework [45]. Computer models of disease progression and treatment response can represent each physical individual (digital twin), or a hypothetical individual whose key characteristics (represented by the inputs of the model) are sampled from the joint distribution of a representative population (digital trials) [46].

Digital twin models can be used to predict how an individual patient will respond in certain conditions.

Digital trials can be used to inform the design of a clinical trial involving physical patients, to provide an early estimate of efficacy over large simulated cohorts, etc. An interesting use of digital patient cohorts is to provide a prior in Bayesian adaptive clinical trial designs [47].

## 5 Conclusion

The aim of this paper was to provide a step-by-step overview of the credibility assessment of predictive biomedical models according to recently published standards and guidelines. While these standards and guidelines were developed to evaluate models used to assess new medical products, we are convinced that the same level of scrutiny should be applied to models used in applied biomedical research.

The ASME V&V 40-2018 standard establishes a solid basis for the credibility assessment of physics-based, mechanistic models used in the regulatory evaluation of new medical devices. However, some caution should be used when evaluating the credibility of other = model types, such as machine learning, grey-box models, or agent-based models.

The credibility assessment of predictive models used for the evaluation of new drugs is currently being discussed. Early guidelines developed by EMA for physiologically based pharmacokinetics models suggest an approach similar to those proposed by ASME V&V 40-2018.

While for regulatory submissions the reference documents are, and must remain, the technical standards and the official guidelines provided by the regulators, we hope this paper can help the research community to better understand, and hopefully more widely adopt, the elements and criteria included in the V&V 40-2018 credibility assessment methodology and the EMA PBPK guideline for establishing and evaluating model credibility. This should ensure that their models are qualified for the intended use even when the modelling activities are not directly or immediately aimed at regulatory approval, such as in peer-reviewed publications.

Computer models can be used for many purposes in biomedical research. But when the predictions of a computer model are used to make clinical recommendations, there is implicit risk to the patient(s) associated with that computer model; and therefore it is necessary to demonstrate the credibility of the model is commensurate with the patient risk [48]. In this sense, we recommend authors, reviewers and editors of peer-reviewed publications to consider at least the key elements of verification, validation, and uncertainty quantification as essential requirements for any publication that relies on mechanistic modelling and simulation.

## Footnotes

1 https://www.fda.gov/regulatory-information/search-fda-guidance-documents/reporting-computational-modeling-studies-medical-device-submissions.

2 https://www.asme.org/codes-standards/find-codes-standards/v-v-40-assessing-credibility-computational-modeling-verification-validation-application-medical-devices.

3 https://www.ema.europa.eu/en/documents/scientific-guideline/guideline-reporting-physiologically-based-pharmacokinetic-pbpk-modelling-simulation_en.pdf.

4 https://www.asme.org/codes-standards/find-codes-standards/v-v-10-guide-verification-validation-computational-solid-mechanics.

5 https://www.asme.org/codes-standards/find-codes-standards/v-v-20-standard-verification-validation-computational-fluid-dynamics-heat-transfer.

6 https://www.fda.gov/combination-products/about-combination-products/combination-product-definition-combination-product-types.

7 https://www.fda.gov/drugs/development-approval-process-drugs/drug-development-tool-qualification-programs.

8 https://www.ema.europa.eu/en/human-regulatory/research-development/scientific-advice-protocol-assistance/qualification-novel-methodologies-medicine-development.

9 https://www.ema.europa.eu/en/documents/scientific-guideline/draft-guideline-qualification-reporting-physiologically-based-pharmacokinetic-pbpk-modelling_en.pdf.

10 https://www.fda.gov/regulatory-information/search-fda-guidance-documents/physiologically-based-pharmacokinetic-analyses-format-and-content-guidance-industry.
